# Warmer Temperature and Spatiotemporal Dynamics during Primary Succession on Tropical Coastal Dunes

**DOI:** 10.3390/plants11223029

**Published:** 2022-11-09

**Authors:** M. Luisa Martínez, Octavio Pérez-Maqueo, Gabriela Vázquez, Rosario Landgrave

**Affiliations:** 1 Red de Ecología Funcional, (Department of Functional Ecology), INECOL, Xalapa-Enríquez 91073, Mexico; 2 Red de Ambiente y Sustentabilidad (Department of Environment and Sustainability), INECOL, Xalapa-Enríquez 91073, Mexico

**Keywords:** primary succession, coastal dunes, Mexico, facilitation nucleation, psammophytes, *Chamaecrista chamaecristoides*, *Schizachyrium scoparium*, climate change

## Abstract

Coastal dunes are sensitive indicators of climate change: it is expected that higher precipitation and warmer temperature will promote vegetation growth and sand stabilization. Alternatively, dunes may become active during severe droughts, which would reduce plant cover and increase sand mobility. Consequently, it is relevant to explore community shifts and self-organization processes to better understand how coastal dunes vegetation will respond to these projected changes. Primary succession allows the exploration of community assembly and reorganization processes. We focused on three environmental variables (bare sand, temperature, and precipitation) and five successional groups (facilitators, colonizers, sand binders, nucleators, and competitors). For 25 years (from 1991 to 2016), species turnover was monitored in 150 permanent plots (4 × 4 m) placed on an initially mobile dune system located on the coast of the Gulf of Mexico. The spatiotemporal dynamics observed during primary succession were consistent with the facilitation nucleation model. As late colonizers grew and expanded, psammophytes became locally extinct. The spatial patterns revealed that ecological succession did not occur evenly on the dunes. In addition, the increased mean yearly temperature during the last decades seemed to be associated with the accelerated increment in plant cover and species richness, which had not been registered before in Mexico.

## 1. Introduction

Coastal dunes are distributed worldwide and host relatively large biodiversity since they occur in many weather regimes. Thus, the vegetation types and plant species are diverse and variable among regions [1]. Because of their local heterogeneity, they are also diverse within regions. In Mexico, for example, coastal dunes cover nearly 800,000 ha, representing 0.04% of the country’s territory [2]. However, this narrow strip holds 9.4% of the Mexican vascular flora (2072 species). Many are uniquely adapted to the coastal dune environment [3]. The biological heterogeneity of the flora from the Mexican coastal dunes is significant, and there are very few similarities among, for example, the Gulf of Mexico, the Caribbean, and the Pacific coasts [4]. Nevertheless, coastal dunes are not just ecologically relevant. The high biodiversity in these environments delivers relevant benefits to society, such as coastal protection, water filtering, nutrient cycling, habitat for endangered species, recreation, and scenic beauty [5,6].

Coastal dunes are also sensitive indicators of climate change. Higher precipitation and/or temperature may promote vegetation growth and increased plant cover. Consequently, coastal dunes become stabilized (fixed) because plants limit aeolian sediment transport. In contrast, the dunes may become active (mobile) when plant cover and dune-stabilizing plants decrease below critical levels during periods of severe drought and evaporation. Alternatively, dunes may also become active or fixed as a result of human intervention (i.e., disturbance, land use changes, and artificial stabilization) [7].

Because of climate change scenarios and predictions, it is relevant to explore community changes and self-organization and assembly processes to better understand how coastal dunes vegetation will respond to these projected changes. This knowledge will be useful to improve management actions that will help preserve this unique vegetation. In this sense, long-term observations of primary succession offer an excellent opportunity to explore community assembly processes because: (a) species richness is locally low, especially on mobile dunes; (b) environmental conditions differ drastically between early and late stages; and (c) changes in community structure and composition take place at a relatively fast rate, especially during the initial colonization period [8,9,10,11].

Primary succession has been historically observed and studied on coastal dunes since the earliest investigations on this topic [12]. In more recent studies, the role of spatial heterogeneity [11,13] and species interactions are acknowledged as essential components of dunes succession. For instance, different studies show that habitat amelioration and nucleation promote the successional process both on mobile dunes [14,15] and in intermediate stages [16]. Early colonizers and facilitator plants improve the environmental conditions promoting colonization by less tolerant species to the mobile dune environment [14,15]. In intermediate conditions, nucleator species [16] again induce the colonization of new species that will eventually constitute more competitive and permanent late-successional stages, including coastal scrubs and even tropical rain forests (in Mexico). As vegetation cover reaches 100%, competition plays a more relevant role throughout the system [17].

More recent studies on the spatiotemporal patterns of ecological succession on coastal dunes have used remote sensing techniques to evaluate long-term forest succession [18]; the recovery of vegetation after the impact of a hurricane [19]; and to explore the impact of urbanization, land use change and human activities [20,21]. Field observations have also shown a substantial alteration of the successional sequence due to the human-induced loss of psammophilous vegetation [22], while Feagin and Wu [10] analyzed spatial patterns of functional groups during succession and used spatially explicit mechanisms. In Mexico, previous studies explored how species replacement between successional stages varied according to local microtopography [11]. The study by Martínez et al. [11] showed that colonization of mobile dunes started in the higher and more mobile parts of the dunes (arms and crests), and from there, the plants expanded toward the inner slope, with reduced dune mobility. Colonizers and facilitator species (psammophytes) started dune stabilization. Later studies explored changes in different diversity measures during succession [8]. Martínez et al. [8] found that the abundance and diversity of psammophytes decreased during succession, whereas species less tolerant to sand movement became dominant. These authors also observed that, over time, diversity changed in a hump-backed manner.

Because of the lack of studies in Mexico, our aim was to explore the impact of environmental changes on the temporal and spatial patterns of ecological succession occurring in a tropical coastal dune plant community. We focused on three environmental variables and five successional groups. The environmental variables included the percentage of bare sand, mean annual temperature, and total yearly precipitation. The first is indicative of the successional process, and the last two are associated with changes in the local weather regime. Successional groups are plant functional groups that were determined based on their ecological role during succession and are specific to coastal dunes [10,16].

To achieve our goal, we first analyzed how environmental variables and vegetation varied over time. Second, because of the relevant role of microtopography on species turnover [11], we looked for spatial patterns during ecological succession and explored if these patterns varied according to the different successional groups. Finally, we looked for statistical correlations between the most dominant plant species and species richness as an indirect estimation of species interactions.

The study took place within a protected area (CICOLMA), in a coastal dune system located along the coast of the Gulf of Mexico, in the state of Veracruz (Mexico). Here, we were able to monitor vegetation changes during a long period, without our study plots suffering any kind of vandalism. To our knowledge, the dunes in the state of Veracruz (and possibly elsewhere in the country) have remained mobile for a long period [23], probably since they were initially formed nearly 3000 years ago [23]. The oldest evidence of the existence of large mobile dunes systems along the state of Veracruz is found in documents and paintings form the colonial period, in the 16th century [24]. According to our observations, the dunes have become gradually vegetated during the recent decades, and this has been associated with changes in the local weather regime [25].

## 2. Results

We observed contrasting changes in biotic and abiotic variables during the 25 years of monitored primary succession sequence. This section shows how bare sand, weather, and vegetation (cover and richness) varied over time and in space.

### 2.1. Temporal Changes during Succession

#### 2.1.1. Bare Sand, Local Weather, and Plant Cover

**Figure 1:**
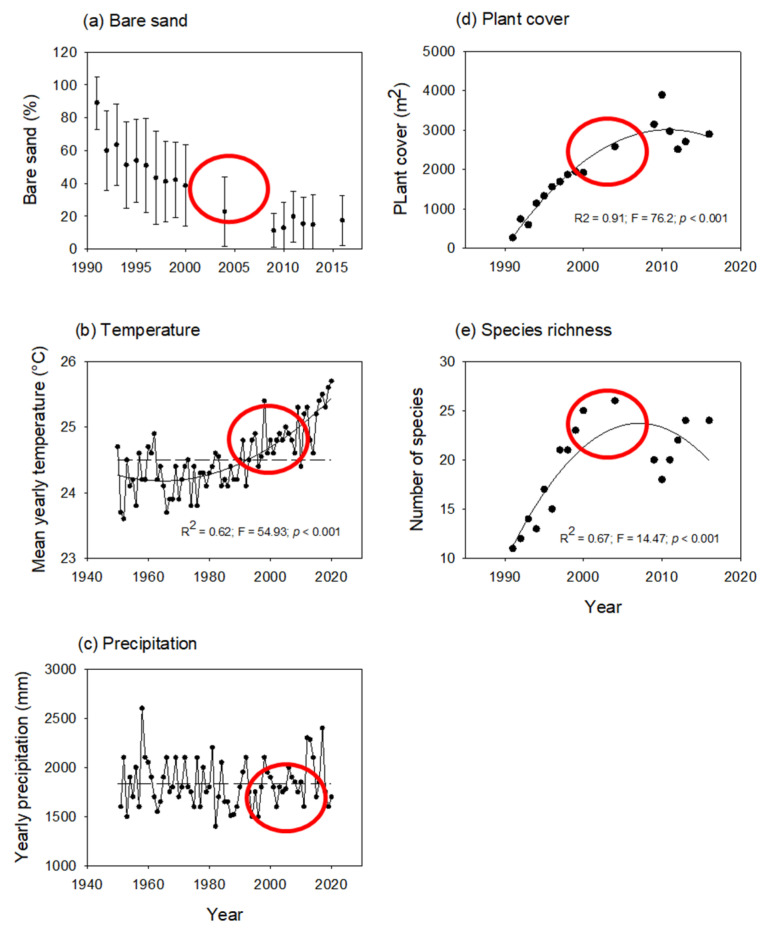
Temporal variability of environmental variables (**a**) mean bare sand per plot (1991–2016), (**b**) total yearly precipitation, (**c**) mean annual temperature (1950–2020) from the on-site weather station, and (**d**) total plant cover and (**e**) total species richness (1991–2016) at the study site. Significant correlations are shown with their corresponding regression values. Horizontal fragmented lines show mean values over the period shown. Red circles show consecutive years that were both warmer and drier and that affected vegetation cover.

The mean percentage of bare sand per plot decreased because plants expanded over the mobile dune area. The variability between plots was high, but the decreasing trend is evident (Figure 1a). The mean percent bare sand remained with minor changes during the latter years, fluctuating between 10% and 20%.

Mean annual temperature and total yearly precipitation varied over time (Figure 1b,c). It is noteworthy that from 2010 to 2015, the mean annual temperature was relatively cooler, and precipitation was relatively dryer; 2004 and 2014 were the driest years in the recorded period. Plant cover and species richness (Figure 1d,e) increased in a humped-back manner over the years, although the largest increment was observed at the beginning of the 2000s, in coincidence with a warmer period.

#### 2.1.2. Successional Groups

**Figure 2:**
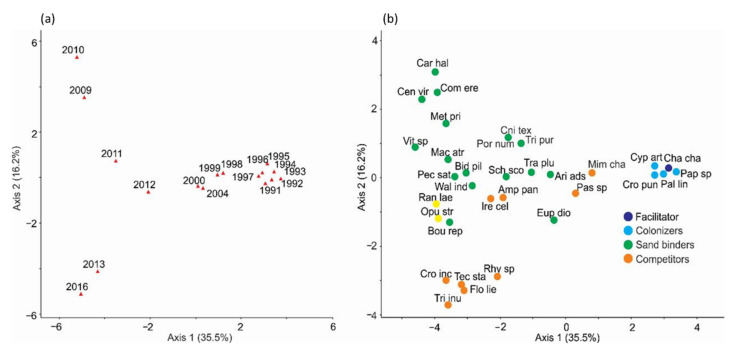
Principal component analysis biplots showing temporal changes in species abundance and community composition over the years during a 25-year successional sequence on mobile coastal dunes in Veracruz, located in the central region of the Gulf of Mexico. (**a**) Site scores; (**b**) species loadings. Red triangles show the location of site scores (years) in the ordination space. Successional groups and complete species names are shown in Table 1.

The principal component analyses using plant cover per species per year during 25 years of observations (1991–2016) suggested a successional sequence in community composition and abundance (Figure 2). Axes 1 and 2 accounted for 35.5% and 16.2% of the total variance (see Supplementary Materials Table S1). The third component was not considered because it only explained 9.6% of the total variance. Figure 2 shows the distribution of years (Figure 2a) and species (Figure 2b) along the two axes. The first ordination axis revealed the successional sequence (Figure 2a). The earlier successional stages appear on the positive end of axis 1 (when bare sand percentage was highest), and mid- and late-successional stages toward the negative end. Colonizing species such as *Palafoxia lindenii*, *Croton punctatus*, *Cyperus articulatus*, *Pappophorum* sp., *Metastelma pringlei*, and *Chamaecrista chamaecristoides* (the only colonizer-facilitator species) had high positive loadings on axis 1 and appeared toward the right-hand side of the plot, as they were more abundant from 1991 to 1997 (Figure S1 and Figure 2a,b). In contrast, sand-binder species such as *Schizachyrium scoparium*, *Walteria indica, Pectis saturejoides, Macroptilium atropurpureum*, and *Bidens pilosa* had high negative loadings on axis 1 and appeared toward the left-hand side of the plot, as they showed high coverage mostly from 2009 to 2016. Other sand binders such as *Commelina erecta, Centrosema virginianum, Cnidoscolus texanus, Triplasis purpurea, Cardiospermum halicacabum, Metastelma pringlei*, and *Porophyllum nummularium*, which predominated in 2009 and 2010, had high positive loadings on axis 2 and appeared toward the upper side of the plot (Figure S1 and Figure 2a,b). The nucleator species, *Opuntia stricta* and *Randia laetevirens*, and competitors such as *Crotalaria incana*, *Florestina liebmannii, Trixis inula*, and *Tecoma stans* had high negative loadings on axis 2, mainly occurred from 2012 to 2016, and appeared toward the bottom part of the plot. A few competitor species such as *Mimosa chaetocarpa* and *Passiflora foetida* were abundant in 1998–1999 and 2000–2004. Bartlett’s test was significant, indicating that there were significant correlations between variables (species coverage in this case), and the null hypothesis of no correlation between species can be rejected.

**Figure 3:**
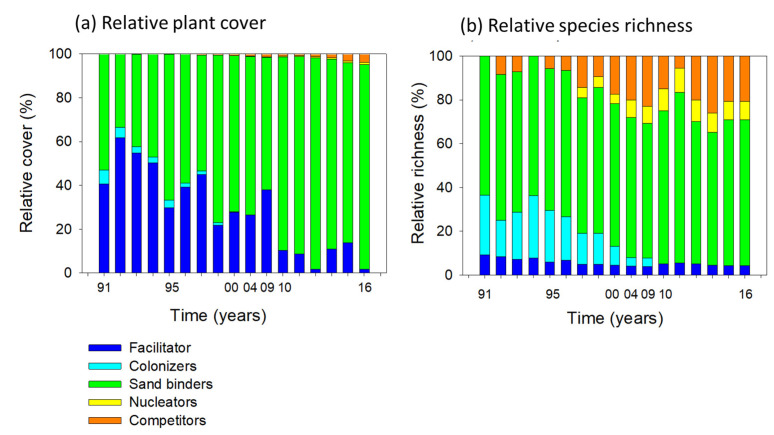
Temporal changes of (**a**) relative plant cover and (**b**) relative species richness during a 25-year successional sequence on mobile coastal dunes in Veracruz, located in the central region of the Gulf of Mexico. Successional groups and species included in each are described in Table 1.

Relative plant cover and relative species richness varied over time among the successional groups. Initially, the facilitator species, *Chamaecrista chamaecristoides*, covered a relatively large area of the studied mobile dunes area, representing 40% to 60% of the total vegetated area (Figure 3a). Gradually, this species became decreasingly abundant as sand binders expanded their cover. The last observed years show that the plant cover by nucleators and competitors has started to increase. The plant cover of the facilitator slightly increased in 2009, 2014, and 2015, but by 2016, it had become almost locally extinct. These trends in plant cover contrast with changes in relative species richness (Figure 3b). Here, the facilitator’s percentage remained relatively unchanged because the only species in this group has decreased in cover but has remained present. The relative number of colonizing species decreased until they became absent in 2010. Sand binders have remained very abundant but unchanged, while species richness of late-successional groups, nucleators, and competitors has increased, especially since 1997 (Figure 3b).

#### 2.1.3. Species Turnover

**Figure 4:**
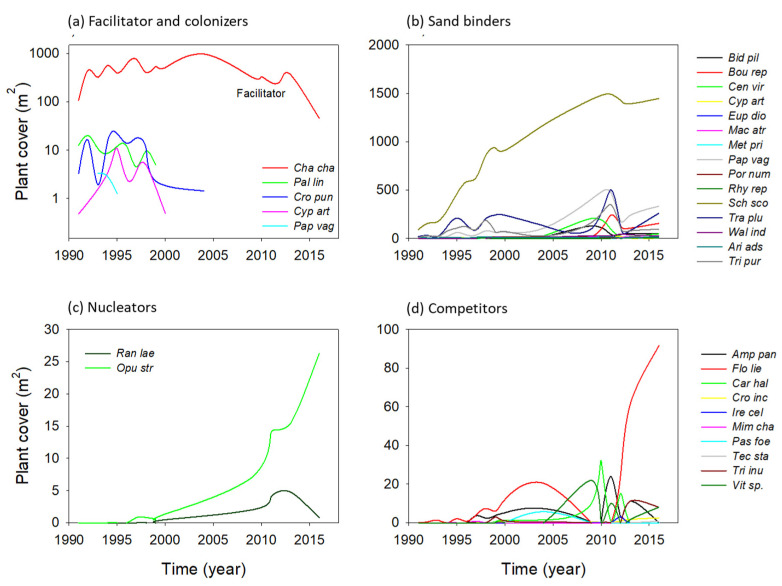
Species turnover during a 25-year primary succession taking place on coastal dunes in the central region of the Gulf of Mexico. Species were grouped according to four successional groups. Scale with plant cover is different for each group for clarity. Table 1 shows full species names. The lines were smoothed for clarity under the supposition that the trends during the missing years were linear. Changes in plant cover of (**a**) facilitators and colonizers (**b**) sand binders; (**c**) nucleators and (**d**) competitors from 1991 to 2016.

Species turnover was very dynamic in all successional groups. Colonizers did not reach high plant cover values and soon became locally extinct. The only colonizer that persisted for the observed period was *C. chamaecristoides*, the species identified as a facilitator (Figure 4a). The number of species identified as sand binders was much higher, but the majority remained relatively scarce most of the time, and only one grass, *Schizachyrium scoparium,* persisted and maintained a high plant cover since 2000 (Figure 4b). As expected, plant cover of nucleators and competitors was reduced during the initial stages and increased in the last observed years (Figure 4c,d). Some species, such as *Florestina liebmannii* and *Passiflora foetida*, became locally extinct in 2010 and then recolonized and became abundant later. Interestingly, we observed that only one species was dominant over the rest for each successional group.

### 2.2. Spatial-Temporal Changes during Succession

**Figure 5:**
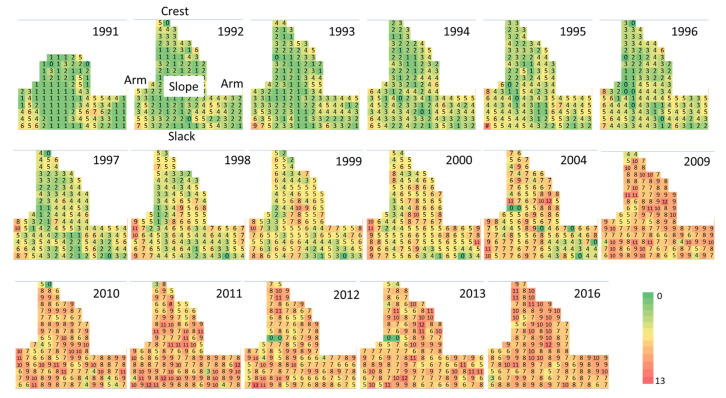
Spatial and temporal patterns of plant species richness during a 25-year successional sequence on mobile coastal dunes in Veracruz, located in the central region of the Gulf of Mexico. Numbers and colors represent species richness. Figures represent the spatial distribution of the plots within the mobile dunes area.

**Figure 6:**
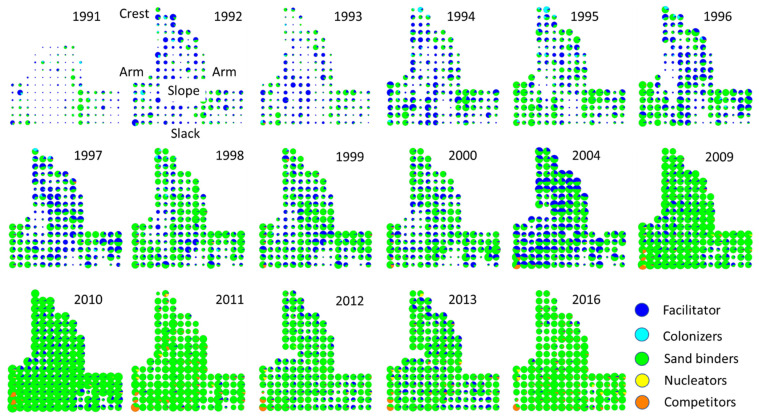
Spatial and temporal changes in the abundance and spatial distribution of successional functional groups turnover during a 25-year successional sequence on mobile coastal dunes in Veracruz, located in the central region of the Gulf of Mexico. Successional groups are described in Table 1. The size of the pie charts represents the total plant cover. The slices in the charts show the relative abundances of successional groups. Figures represent the spatial distribution of the plots within the mobile dunes area.

The successional sequence did not occur homogeneously on the monitored mobile dune (Figure 5 and Figure 6). Species richness and functional groups were distributed heterogeneously throughout the monitored area. Species richness was initially low, although higher on the arms. The number of species increased slowly, from the outer section of the mobile dunes area (dunes and crest), toward the center (the slope) (Figure 5). The plots with the highest number of plant species were located near the slack and on the arms and crest. In later years, the number of plant species per plot was more homogeneous throughout the study site. Small-scale disturbances occurred in some plots where species became locally extinct, and the plant cover was lost, especially in 2004. These changes were possibly the result of a sequence of drier and warmer years from 2000 to 2004 and of small-scale trampling and herbivory by local fauna, such as rabbits. Plant species richness per plot was relatively high in the late-successional stages in 2016.

Like species richness, plant cover was initially scarce throughout the monitored area, as observed by the reduced size of the pie charts in Figure 6. Dune colonization and stabilization occurred from the arms toward the center (the slope). The facilitator species was initially present on the arms and expanded toward the slope, where minimal vegetation cover was present. In contrast, the other colonizers were mainly found on the dune’s crest and arms (Figure 6). Like the facilitator, sand binders colonized and expanded their cover first on the arms and then on the slope until they became dominant in 2009. Nucleators and competitors arrived on the arms, closer to the slack, from where they are gradually increasing their abundance. It is noteworthy the change observed in 2004, when the abundance of the facilitator increased noticeably, although this trend had reversed by 2009.

**Figure 7:**
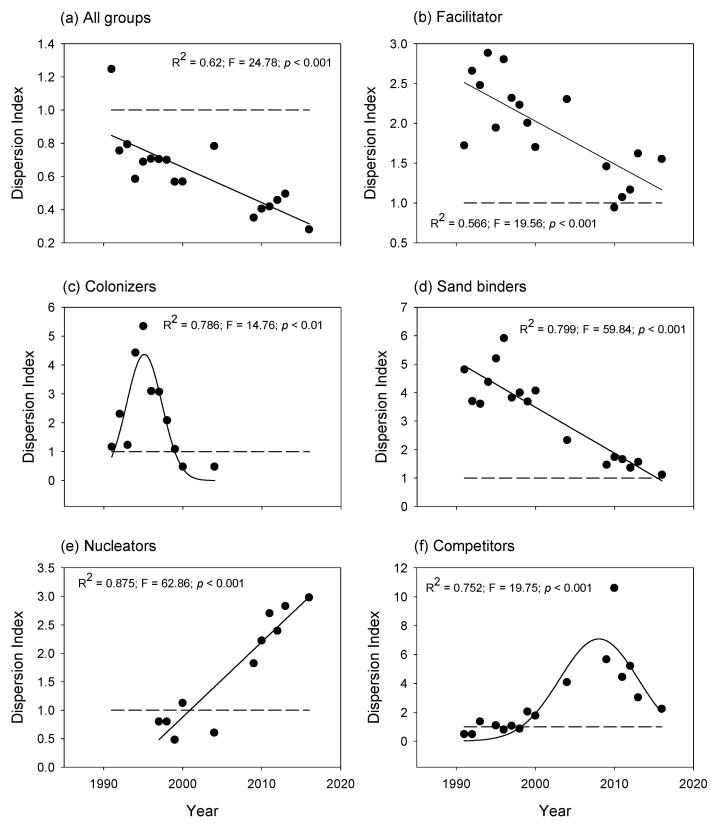
Dispersion index (D.I.) calculated for (**a**) all species, independently of their successional group; and then for each successional group, (**b**) facilitators; (**c**) colonizers; (**d**) sand binders; (**e**) nucleators and (**f**) competitors. When D.I. > 1, the distribution is aggregated; D.I. ≈ 1, the distribution is random, and when D.I. < 1, the distribution is regular [26]. Successional groups are described in Table 1. Dotted lines show D.I. = 1 for reference.

The dispersion index calculated for all groups and then for each successional group helps observe changes in the spatial aggregation of species. When species are considered independently of their successional group (Figure 7a), the dispersion index is only > 1 initially, and then the remaining values are <1. This means that the species in the plant community were initially aggregated, and then the spatial distribution was regular. The same linear negative trend was observed for the facilitator (Figure 7b), but the dispersion index was >1 most of the time, indicating a predominant aggregated pattern. Over time, this dispersion index reached values closer to one, which meant a relatively random distribution. The dispersion pattern of the colonizers was not linear but had a peak in the late nineties (Figure 7c). Here, the colonizers seemed randomly distributed at the beginning and end of the observed period, when the dispersion index values fluctuated around 1, but were aggregated during the nineties. The dispersion patterns of the sand binders were like the facilitator, but the aggregation values were much higher (Figure 7d). The dispersion index of the nucleators was very different from the other groups (Figure 7e) because it started with a nearly random pattern (dispersion index equal to one) and then gradually became more and more aggregated, shown by dispersion index values >1). Finally, the competitors had an initially random distribution, which then became aggregated (Figure 7f), reaching a peak in 2010. The dispersion index of the competitors decreased during the most recent years, meaning that the dispersion became increasingly random. Could we have less space beneath this paragraph? 

### 2.3. Species Interactions: Facilitators and Sand Binders vs. Species Richness

Our results showed an evident dominance of three species, the facilitator (*C. chamaecristoides*), one sand binder (*S. scoparius*), and the nucleator (*O. stricta*) (Figure 4). Even though *F. liebmannii* was more abundant than *O. stricta*, we chose to focus on the latter because previous studies indicate that this species acts as a nucleator species at the study site, whereas there is no such information for *F. liebmannii* [27].

**Figure 8:**
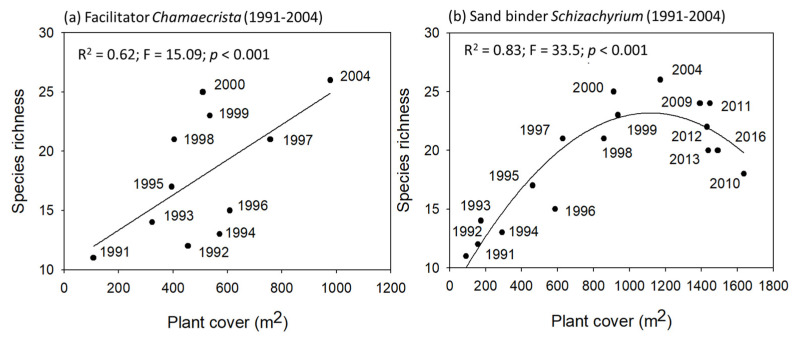
Correlation analyses between plant cover of the two most abundant species in the primary succession sequence and species richness. (**a**) *Chamaecrista chamaecristoides* is a facilitator from initial successional stages, and (**b**) *Schizachyrium scoparius* is a sand binder typical of intermediate stages. Black dots represent plant cover values of either the facilitator (**a**) or the sand binder (**b**), respectively, and the total species richness observed each year.

We explored the potential interaction between these dominant species and plant species richness by performing correlation analyses, and a statistical model fit that would provide indirect information on the impact of these species in community dynamics during primary succession. We found a linear relationship between the facilitator and species richness from 1991 to 2004 (Figure 8), when the abundance of *C. chamaecristoides* started to decline (data not shown). The relationship between the sand binder and species richness was not linear but quadratic. Initially, species richness increased as the grass *S. scoparius* expanded its cover. However, in 2004 this trend reversed, and species richness began to decline while the grass continued growing. These results are indirect evidence of facilitative and competitive interactions, respectively. Finally, no clear trends were observed between the nucleator, *Opuntia stricta*, and plant species richness (plot not shown).

## 3. Discussion

The aim of this study was to explore the impact of environmental changes on the temporal and spatial patterns of ecological succession occurring in a tropical coastal dune plant community. We focused on three environmental variables (bare sand, temperature, and precipitation) and five successional groups (facilitators, colonizers, sand binders, nucleators, and competitors). Our results show that:

(a) Temporal trends—As expected during any successional process on coastal dunes, the percentage of bare sand declined over time. We also observed changes in the local weather regime with an increased mean yearly temperature during the last decades. In turn, plant cover and species richness changed in a hump-backed manner. The increment in plant cover and species richness coincided partially with the increased temperature.
(b) Spatial patterns —The facilitator, colonizers, and sand binders grew first on the arms, slope, and crest. In turn, the competitors and nucleators grew on well-developed dune ridges and near the slack, with dense plant cover. In addition, the dispersion index showed that the spatial patterns of plant species distribution were aggregated during different periods of the 25-year successional sequence.
(c) Species interactions —The statistical correlations between the most dominant plant species and species richness was used as an indirect estimation of species interactions. Species richness increased linearly with increasing plant cover of the facilitator, showing indirect evidence of facilitation. In turn, the grassy competitor seemed to inhibit the colonization of new species beyond a threshold plant cover.

### 3.1. Temporal Trends

The decreased percentage of bare sand and the increment in plant cover and species richness have been observed in other coastal dune systems and are referred to as the “greening” of coastal dunes [28]. Causal explanations of this ecological trend are complicated because there is not a single variable that solely and directly affects plant succession and vegetation cover.

In this study, and in coincidence with previous findings [28], the increased plant cover and species richness were associated with warmer mean annual temperatures. Nevertheless, a growing plant cover in coastal dunes has also been associated with higher precipitation in Brazil [29,30] and Canada [31]; changing wind regimes in Canada, Brazil [29,31,32], and Israel [33]; and longer growing seasons in Ireland [34]. Some authors also found multifactorial causes that explain the stabilization of European coastal dunes: changes in land use, crashing rabbit populations, eutrophication, climate change (higher temperatures and precipitation), the expansion of invasive species, artificial stabilization actions, and less windy conditions, [35,36]. Apparently, there is a global trend of sand dune stabilization, mostly associated with climatic changes [28]. These trends had not been previously reported in historical records of Mexico (1500s) that mention the existence of mobile dunes in Veracruz, for example [24].

The increased temperatures during the recent decades seemed to be associated with the higher relative cover of sand binders and nucleators and the greater species richness of competitors. In our study site, *Schizachyrium scoparium* is the dominant grass among the sand binders, with a C4 photosynthesis pathway [37] which is associated with warmer climates [38,39]. Thus, a warmer climate would be beneficial for these C4 species. Additionally, the nucleator species *Opuntia stricta* var. *dilenii* and *Randia laetevirens* [27] are native to Mexico and are found in very warm habitats. *Opuntia* grows best in dry arid conditions, whereas *Randia* is mostly found in more humid tropical ecosystems such as tropical forests and coastal thickets [40,41,42]. Consequently, it is no surprise that these plants increased their cover with warmer temperatures. Finally, the species included in the competitor group are abundant in tropical weather, and thus, warmer temperatures seemed to be favorable for them.

In contrast with the above-mentioned findings and trends, other studies revealed that dune vegetation in Scotland [43] appeared to be resistant to climate change, and no clear geographical patterns (for instance, increased dominance of more southerly species in northern latitudes) were observed. Nevertheless, a general greening also was observed in the Scottish dunes. Indeed, studying the complexities of the impact of climate change on dune vegetation needs further detailed studies, the comparison between different sites, and the consideration of different variables, which may act as drivers.

### 3.2. Spatial Patterns

Changes in spatiotemporal patterns during succession on coastal dunes have attracted considerable attention for over 100 years [12]. Since then, primary succession on coastal dunes has been studied in many parts of the world. Species turnover is closely associated with the spatial patterns of plant distribution and environmental heterogeneity [10,16,18].

Possibly, the spatial distribution of the dune species is associated with a combination of variables such as water availability [44] with less arid conditions in the lower sections of the dunes [45]. This results in drought-tolerant species (i.e., colonizers and facilitators such as *Chamaecrista chamaecristoides*) growing on the arms and crests where the phreatic level is deeper. In turn, the least drought-tolerant species are found in the arms and dune slacks (i.e., the sand binders and competitors: *Schizachyrium scoparium* and *Florestina liebmannii*). Additionally, sand mobility may also affect the spatial distribution of plants. Like drought, species tolerant to substrate mobility (colonizers and facilitators) are found on the most mobile areas within the dune system (frequently the arms and crests) [45], whereas the competitors grow on the more stable dunes near the slacks and the inner slope. Inter- and intra-specific interactions are also known to affect the spatial distribution of plants [46], but these have not been explored in our study site.

The increment in species richness and the number of functional groups started on the arms and crest of the dunes and then continued toward the center, on the slope. This pattern also was found in previous studies by Martínez et al. [11], who equally reported that species turnover rates varied between locations within a mobile dunes area. Species replacement first occurred on the arms, then on the crest, and finally on the slope. Like in temperate coastal dunes [10,18,20,47], primary succession in tropical latitudes is spatially and temporally heterogeneous.

The index of dispersion showed different spatial arrangement patterns of the successional groups. Interestingly, the aggregation index calculated for each successional group differed from that calculated for all plant species, independently of their successional role. In this case, the dispersion index only revealed an aggregated pattern during the first observed year, and then the spatial distribution was regular. The pattern in which a trend appears in several groups but disappears or reverses when the groups are combined is known as Simpson’s paradox [48]. This paradox has several statistical implications, but in our case, it shows that the whole does not represent the parts, and thus, it is relevant to explore spatial trends based on the successional groups. This is especially important when studying community assembly and self-organization patterns.

### 3.3. Successional Groups and Species Interactions

Conceptual models on primary succession typically predict several phases: initial colonization during extremely harsh conditions, frequently characterized by the occurrence of psammophilous species acting as facilitators. After the initial mobile dune colonization, a nucleation process occurs with patches promoting again the establishment of later successional species (persistent or competitor species) that will eventually constitute coastal thickets and forests [16,47]. The successional groups observed in this study also occur on coastal dunes elsewhere, for example, Texas (USA) [10], The Netherlands [18], Italy [22], and Spain [20].

The paradigm of facilitation–competition under the stress-gradient hypothesis has received attention for the last decades [17]. Facilitation is most common in severe environments where plants are exposed to high levels of physiological stress and benefit more from the microclimate amelioration effect [49]. Previous studies mention a switch in the net interactions from facilitation to competition with decreasing environmental stress [50,51,52]. In our case, in different field experiments, Martínez [14] and Martínez et al. [15] demonstrated spatial aggregation of grass species beneath the shade of the facilitator (*C. chamaecristoides*). Furthermore, the above-mentioned studies also demonstrated that the amelioration of environmental conditions improved the survival of seedlings of colonizer and sand binder species beneath the shade of the facilitator. The role of the facilitator in community composition was observed indirectly in this study by the increasing richness as the plant cover of the facilitator expanded.

Evidence on competition is indirect as there is no experimental data. The correlations between plant cover of the most abundant grass, *S. scoparius*, and species richness showed an asymptotic increment in species richness, suggesting a competitive interaction. Nevertheless, rigorous testing is necessary to confirm this. An integrative and mechanistic approach is needed to test the facilitation–competition paradigm.

## 4. Materials and Methods

### 4.1. Study Site

**Figure 9:**
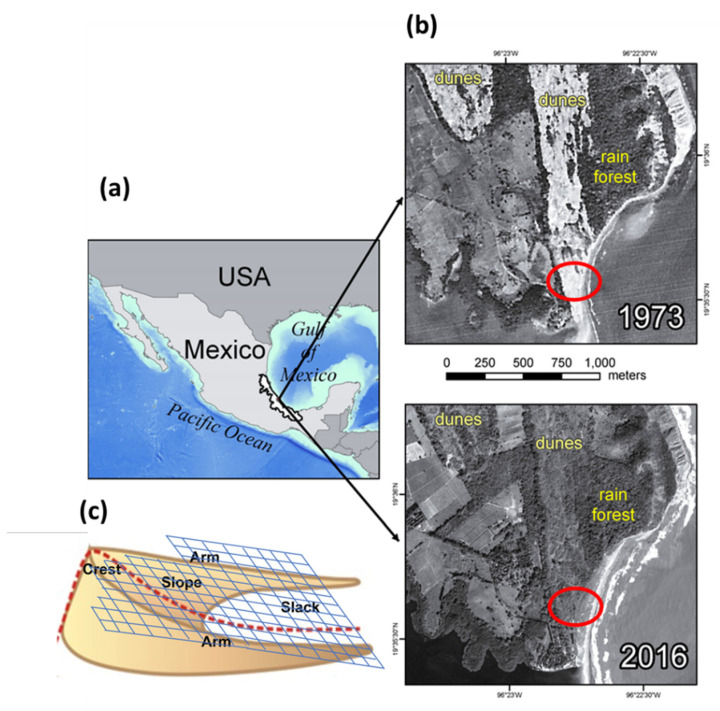
(**a**) Location of the study site. (**b**) Aerial images showing the initial mobility of the coastal dunes. Red circles show the approximate area where the permanent plots were placed for long-term monitoring. (**c**) The location of the grid and topographic elements where the sampled plots were placed. Red circles show the location of the study site within the reserve.

The research took place at the Centro de Investigaciones Costeras La Mancha (CICOLMA) (Center of Coastal Investigations La Mancha), located in the state of Veracruz on the coast of the Gulf of Mexico (19°31′ N, 96°23′ W) (Figure 9). Parabolic north-south oriented dunes that may reach up to 20 m in elevation surround the beach at CICOLMA. Initially, these dunes had different degrees of stabilization. The dune system extends over 2 km along the coast; it borders a coastal lagoon to the south and a fossil dune to the north. Mean annual precipitation is 1260 mm, most of it occurring during the summer months (June to October) [11]. The dry season takes place from November to May and coincides with the occurrence of cold fronts with strong northerly winds (>80 km/h) and intense sand movement [11].

In the past, the coastal dune system at La Mancha was highly mobile, as can be observed from aerial photographs (Figure 9) dating from 1973. The parabolic and transverse dunes at the site are recent sand deposits running from north to south. They were built during the small regression period after 3500 B.P. [23]. Gradually and naturally, the shifting sand was colonized by native vegetation until the dunes became stabilized during the last decades. The recent stabilization of the dunes has been associated with climate changes, with increased precipitation and warmer temperatures in the last decades. These trends are like those observed in nearby dune systems [8].

### 4.2. Environmental Variables

We monitored three environmental variables: percent bare sand, temperature, and precipitation. We estimated the percent bare sand to assess how the mobile dunes area became colonized by vegetation. To achieve this, we visually estimated the percent bare sand in each quadrat on each occasion when we sampled vegetation.

In addition to bare sand, we gathered long-term (1983–2020) information from the local weather station located at La Mancha, from where we obtained mean yearly temperature and total yearly precipitation.

### 4.3. Vegetation Sampling

In 1991, we chose a mobile dunes area (56 × 60 m) located 280 m inland from the beach and surrounded by a heterogeneous environment that included mobile dunes covered by sparse grasslands, coastal thickets, and tropical rainforest (Figure 9). In general, the coast at the study site is accumulative. However, during the study period, we observed changes in the width of the beach, with severe erosion occurring during the early 1990s owing to intense hurricane activity and beach progression beginning in the 2010s. Nevertheless, the distance from the monitored site to the back of the beach, where the coastal dunes begin to develop, has not changed. This was confirmed by measuring the distance from aerial pictures, which remained at 280 m on both dates.

In 1991, at the beginning of our study, the vegetation on the mobile dunes was sparse and thus represented the earliest successional stages. We established 140 permanent plots (4 × 4 m) for monitoring purposes, forming a grid covering the selected mobile dunes area. This method has proved helpful because it allows the mapping and location of plants and guarantees that the area remains undisturbed for a long time, especially in a preserved site such as La Mancha.

Each plot was marked with 1 m long aluminum stakes, driven into the sand (50 cm buried and 50 cm exposed), and located in all four corners. We identified and recorded every vascular species in each plot and visually estimated the percent cover per species [8]. As plants grew and expanded their cover, the added percent cover per plot increased and eventually surpassed the 100% value because they overlapped on different layers.

All plots were monitored at different time intervals at the end of the rainy season, in October, when plant cover is highest. Vegetation was recorded annually from 1991 to 2000, in 2004, from 2009 to 2013, and finally, in 2016. The total period of observations reached 25 years.

### 4.4. Successional Groups

Plant species observed in each plot were placed into successional groups based on groups obtained from field observations and literature review [34] and supported by a subsequent principal component analysis (see below). We applied the classification system of Feagin and Wu [10], who used colonizers, sand binders, and competitors. Transferring the same groups from the coastal dunes in Texas [10] to our study site is viable because the successional sequence on coastal dunes is similar among systems, even though the species involved in the process are different. In addition to the above-mentioned successional groups, we included two new ones: facilitators (one species) [14] and nucleators (two species) [16]. Both Moreno-Casasola 1988 [42] and Yarranton and Morrison [16] showed how these species ameliorate harsh environmental conditions and, thus, promote the colonization and establishment of less tolerant species (Table 1). Successional groups are defined as follows, based on Gallego-Fernandez and Martínez [53] and Yarranton and Morrison [16] (a) colonizers —plant species that are typical of mobile dunes that only establish in these habitats; (b) facilitators —plant species that are typical of mobile dunes and that ameliorate environmental conditions promoting the colonization of less tolerant species; (c) sand binders —plant species from semi-mobile dunes (with less substrate mobility) that are moderately tolerant to sand mobility; they fix sand within their root systems; (d) nucleators —species not tolerant to sand burial, typical of stabilized dunes; they modify environmental conditions and promote the colonization of species not tolerant to intense sunlight and high temperature; nucleators were shrubby species; (e) competitors —plant species typical of stabilized coastal dunes and inland communities, not tolerant to sand burial; these are considered to be competitive since they grow in high diversity sites. Competitors are mostly shrubs and trees. The groups used are like the competitor-stress-ruderal classification system of Grime [54] but are adapted to the dune environment. Species grouped in the “stress” category are like facilitators and colonizers; ruderals could be equivalent to sand binders. However, it is important to note that our definitions are relative to the context of coastal dunes.

**Table 1:** Successional groups and plant species from the coastal dune community on La Mancha coastal dunes, Veracruz, Mexico. Successional groups were identified from field observations and literature review [34] and supported by a subsequent principal component analysis. The most abundant species are shown for each group. In addition, plant attributes and typical locations within the dune system are presented for each functional group.

Successional Group	Plant Species	Attributes	Typical Location
Facilitator	*Chamaecrista chamaecristoides*	Ameliorates harsh conditions	Mobile dunes
Colonizers	*Palafoxia lindenii*, *Croton punctatus*, *Cyperus articulatus*, *Pappophorum vaginatum*	Tolerant to burial by sand	Mobile dunes
Sand binders	*Schizachyrium scoparium*, *Trachypogon plumosus*, *Pectis saturejoides*, *Aristida adscensionis*, *Bidens pilosa*, *Bouteloa repens*, *Centrosema virginiana*, *Cnidosculus texanus*, *Commelina erecta*, *Euphorbia dioica*, *Macroptilium atropurpureum*, *Metastelma pringlei*, *Porophyllum nummularium*, *Rhynchelytrum* sp., *Triplasis purpurea*, *Walteria indica*	Less tolerant to burial; better competitors	Semi-mobile dunes
Nucleators	*Randia laetevirens* *Opuntia stricta*	Provide shade, organic matter	Stabilized dunes
Competitors	*Florestina liebmannii* *Tecoma stans* *Trixis inula*, *Amphilopium paniculatum*, *Cardiospermum halicacabum*, *Crotalaria incana*, *Iresine celosia*, *Mimosa chaetorcarpa*, *Passiflora foetida*, *Tecoma stans*, *Vitis* sp.	Efficient competitors, shade tolerance	Stabilized dunes Coastal thickets

### 4.5. Data Analyses

#### 4.5.1. Bare Sand, Temperature, Precipitation, Species Richness, and Plant Cover

We transformed bare sand and species percent cover into squared meters [8,11]. As individual plants grew and expanded over time, they began to overlap. Consequently, the total area covered per species increased until the total vegetation cover became more extensive than the total area in each plot. Mean bare sand per plot and standard deviation were calculated for each year. Correlation analyses of temperature, precipitation, species richness, and plant cover vs. time were performed to look for temporal patterns during the observed period.

#### 4.5.2. Successional Sequence and Species Interactions

We performed a principal component analysis (PCA) to identify the changes in community composition over time during the primary succession sequence. The PCA analysis was applied to the correlation matrix of species coverage (variables) each year using the PCORD program [55]. A Bartlett’s test of sphericity was applied to evaluate the significance of the PCA’s results. Bartlett’s test was significant (*p* < 0.0001), indicating that there were significant correlations between variables (species coverage in this case), and the null hypothesis of no correlation between species can be rejected. A Variance Maximization (varimax) rotation was applied. Each species was assigned to a successional group and identified in the plot with different colors. Then, two Euclidean biplots were constructed, using the scores of the years and the species loadings as coordinates.

Spatial patterns were plotted based on the coordinates we had for each sampled quadrat and the information on each successional group’s plant cover and species richness.

Then, two correlation models were fit between plant species richness and plant cover of the facilitator, *Chamaecrista chamaecristoides*, and the over-abundant sand binder, *Schizachyrium scoparium*. We used the models as proxies, indicative of facilitation and competition, which were driven by these very abundant species.

Finally, we analyzed the dispersion patterns of species and successional groups to explore spatial changes during the successional sequence. Dispersion patterns can be characterized through the index of dispersion, which is the ratio between the variance (s^2^) and the mean number of individuals (or species in our case) $\overset{-}{X}$ within a sampled landscape. As the null hypothesis, it is assumed that species are randomly distributed, and thus, it is expected that the variance is equal to the mean, so the ratio is s^2^/ $\overset{-}{X}$ ≈ 1. When the ratio is >1, it indicates that the population has a clumped distribution because the variance is larger than the mean. The contrary occurs when the index is <1, which would be the result of a small variation between the sampled units and, thus, indicates an even or regular distribution [26].

## 5. Conclusions

Our findings reveal that: (a) species turnover followed the classic successional sequence of coastal dunes: colonizers, facilitators, sand binders, nucleators, and competitors; (b) the warmer mean yearly temperature during the last decades seems to be associated with the increment in plant cover and species richness, but other factors (such as changing wind regimes) may also be relevant drivers behind these trends; (c) spatial patterns appear to affect ecological succession, and these changes depend on the successional groups, and the tolerance of plants to drought and sand movement; (d) facilitation and competition occur during different stages of the successional sequence. The study of the spatiotemporal patterns of primary succession helps in understanding the mechanisms of community development and assembly rules and how they vary spatially according to environmental variability. The above is relevant, especially given the high environmental degradation of coastal dunes and the urgent need for restoration actions. In addition, it is useful to predict potential modifications of community dynamics under climate change scenarios.

## Data Availability

Data are available upon request to the authors.

## Supplementary Materials

The following supporting information can be downloaded at: https://www.mdpi.com/article/10.3390/plants11223029/s1. S1. Supplementary material 1. Results from the Principal Components Analyses, showing eigenvalues, variance percentages, and species loadings on the first two principal components. The species with the highest loadings are also highly correlated with the corresponding component (shown in boldface).
