# Supplementary material for: Warmer Temperature and Spatiotemporal Dynamics during Primary Succession on Tropical Coastal Dunes
Source: Plants (Basel). 2022 Nov 9;11(22):3029. doi: 10.3390/plants11223029 (PMC9697752; doi:10.3390/plants11223029)
Supplement: Supplementary file 1 [file plants-11-03029-s001.zip › plants-1984903-supplementary.pdf]

Supplementary Materials:

S1. Supplementary material 1. Results from the Principal Components Analyses, showing eigenvalues, variance percentages, and species loadings on the first two principal components. The species with the highest loadings are also highly correlated with the corresponding component (shown in boldface).

	Axis 1	Axis 2		
Eigenvalues	11.715	5.347		
Percentage	35.501	16.203		
Cum. Percentage	35.501	51.703		
	PCA variable loadings		Pearson correlations	
	Axis 1	Axis 2	Axis 1	Axis 2
Cha cha	**0.126**	0.049	0.429	0.116
Cyp art	**0.131**	0.037	0.448	0.086
Cro pun	**0.208**	0.034	0.711	0.077
Pal lin	**0.238**	0.02	0.811	0.043
Pap sp	**0.127**	0.013	0.436	0.032
Ari ads	-0.127	0.034	-0.426	0.082
Bid pil	**-0.202**	0.005	-0.694	0.013
Bou rep	-0.189	-0.151	-0.647	-0.349
Cen vir	-0.217	**0.251**	-0.747	0.577
Cni tex	-0.142	0.215	-0.485	0.499
Com ere	-0.196	**0.273**	-0.669	0.632
Eup dio	-0.022	-0.174	-0.079	-0.405
Mac atr	**-0.236**	0.08	-0.81	0.187
Met pri	**-0.213**	0.204	-0.727	0.474
Pec sat	**-0.272**	-0.015	-0.932	-0.029
Tri pur	-0.153	**0.259**	-0.525	0.598
Por num	-0.144	**0.207**	-0.492	0.483
Rhy sp	-0.123	-0.328	-0.422	-0.755
Sch sco	**-0.273**	0.016	-0.936	0.037
Tra plu	-0.131	0.042	-0.453	0.094
Wal ind	**-0.265**	-0.001	-0.906	-0.004
Amp pan	-0.103	-0.065	-0.354	-0.151
Car hal	-0.159	**0.271**	-0.54	0.624
Cro inc	-0.175	**-0.313**	-0.598	-0.724
Flo lie	-0.152	**-0.342**	-0.524	-0.797
Ire cel	-0.046	-0.03	-0.157	-0.07
Mim cha	0.061	0.004	0.209	0.009
Pas sp	0.008	-0.022	0.025	-0.05
Tec sta	-0.102	**-0.234**	-0.35	-0.542
Tri inu	-0.142	**-0.318**	-0.483	-0.731
Vit sp	-0.173	0.074	-0.593	0.172
Opu str	**-0.251**	-0.165	-0.859	-0.377
Ran lae	-0.19	-0.079	-0.656	-0.186

Bartlett´s test sphericity $\chi 2 = 13031$, df= 528, $p<0.000$
